# Incidence of depression, anxiety and stress following traumatic injury: a longitudinal study

**DOI:** 10.1186/s13049-015-0109-z

**Published:** 2015-03-28

**Authors:** Taneal A Wiseman, Kate Curtis, Mary Lam, Kim Foster

**Affiliations:** Sydney Nursing School, University of Sydney, Camperdown, 2050 NSW Australia; Trauma Service, St George Hospital, Kogarah, 2217 NSW Australia; Faculty of Health, University of Canberra, Canberra, 2617 ACT Australia

**Keywords:** Depression, Anxiety, Stress, Injury, Mental health, Patient outcome, Screening

## Abstract

**Background:**

Traumatic injury and mental health disorders are co-associated. Early identification of depression, anxiety and stress following injury, and subsequent preventive intervention, may reduce the long-term symptoms and negative impacts associated with depression and anxiety. The purpose of the study was to determine the incidence, severity and predictors of depression, anxiety and stress in injured patients in the acute phase of care, and at six months following injury, as well as the effectiveness of an in-hospital screening tool.

**Methods:**

This descriptive longitudinal study of trauma patients was conducted at a Level 1 Metropolitan Trauma Centre in Australia over 14 months. Participants were interviewed using the Depression Anxiety Stress Scale short-form version (DASS-21) during hospital admission then at 3 and 6 months after injury. Descriptive statistics were performed to evaluate participant characteristics and incidence of depression, anxiety and stress. Correlations and logistic regression were conducted to investigate the ability of the DASS-21 to predict symptoms of depression, anxiety and stress and to investigate factors associated with depression, anxiety and stress 6 months after injury.

**Results:**

201 participants ranging in age (18–94 years) and injury severity participated in the baseline interview and 109 completed all 3 interviews over 6 months. Over half (54%) reported above normal scores for depression, anxiety and/or stress in at least one of the 3 time points. Intensive care unit admission and high levels of depression, anxiety and stress at 3 months post injury were predictors for high levels of depression, anxiety and stress at 6 months. Low scores for depression, anxiety and stress during admission were correlated with low scores for depression, anxiety and stress at 3 and 6 months.

**Conclusion:**

Depression, anxiety and stress in patients hospitalised following injury is common and should be anticipated in patients who have had an intensive care admission. Screening at 3 months following injury identifies patients at risk of long-term symptoms of depression, anxiety and stress.

## Introduction

Traumatic injury is responsible for 11% of global mortality and contributes to a significant amount of physical and psychological morbidity for all age groups [1]. Patients with traumatic injury report a substantial reduction in health-related quality of life compared to other patients, including long-term psychological and physical disability [2]. The psychological impact of injury includes the development of acute and long-term mental health problems such as post-traumatic stress, depression and anxiety.

Posttraumatic stress disorder (PTSD) is the most frequently researched mental health problem following injury. Acute stress disorder (ASD) [3] can progress to PTSD if symptoms persist after one month with approximately 50% of those with PTSD initially presenting with ASD [4]. Acute stress disorder occurs in up to 45% of injury survivors [5], and involves an anxiety response that includes re-experience of the traumatic event, intrusive memories, dreams, and strong emotional distress on exposure to triggering events [4].

Anxiety has also been found to occur in injury survivors [1,6], with anxiety identified in up to 40% of injury survivors [7-9]. Some characteristic symptoms of anxiety include; hyper arousal and physical symptoms such as increased respirations and dry mouth. Depression has been identified in up to 42% of injury survivors, from as early as 6 weeks to as long as 20 years post injury [8,9]. Characteristic symptoms of depression include feelings of prolonged unhappiness, lethargy and a general lack of interest in the environment and self [3,6].

Despite the known associations between injury, depression, anxiety, ASD and PTSD, there is limited knowledge on the combined presence and extent of depression, anxiety and stress symptoms in injured patients over time [7]. Further, there is little evidence on effective early screening tools, and the implementation of screening tools, for these problems in the injured population [7-10]. The purpose of the study was to determine the incidence, severity and predictors of depression, anxiety and stress in injured patients in the six months following injury and use the findings to inform strategy for effective psychosocial follow-up care of injured patients.

### Aims of the study

The aims of the study were to:

- Determine the incidence and severity of Depression, Anxiety and Stress post injury, during hospital admission and at 3 and 6 months following admission.
- Identify factors associated with higher levels of depression, anxiety and stress in patients post injury.

## Patients and methods

### Design and setting

This was a descriptive longitudinal quantitative study. Following ethics approval (HREC/10/SGH/198), data were collected over 14-months from May 2011 to August 2012 at a Level 1 Trauma Centre in Australia. Depression, anxiety, and stress levels were determined during a structured interview at 3 time points; during hospital admission, 3 months, and 6 months post injury.

### Sample size

Calculations for the primary analyses were based on a repeated measures analysis of variance exploring change over time between the three injury severity groups on DASS-21 subscales (main and interaction effects). With 3 time periods, a medium effect size f of 0.25, alpha of 0.01 (to account for multiple testing) and 80% power, a total sample size of 150 was required. Additional participants were recruited (n = 201) to account for the loss to follow-up associated with longitudinal research.

### Participants

All patients aged 18 years and over admitted to the study site meeting trauma admission criteria were screened for inclusion irrespective of injury mechanism, severity of injury or background. Trauma admission criteria are based on mechanism of injury, physiologic and injury criteria. For example, motor vehicle collision (MVC) greater than 55kph, assault, or fall from height > 3 metres. Patients were excluded if they had a pre-existing cognitive impairment such as dementia or traumatic brain injury resulting in existing cognitive impairment. All patients included in the study provided written consent prior to the collection of any data.

### Tools

The study site trauma registry was used to obtain demographic, injury and outcome data. Participant levels of depression, anxiety and stress were assessed using the short form version of the Depression Anxiety and Stress Scale (DASS-21) [11].

The DASS 21 is a validated self-report tool consisting of three 7-item subscales, assessing the related negative emotion states of depression, anxiety and stress, using a four-point Likert scale ranging from 0–3 (Never, Sometimes, Often and Almost Always) to indicate the frequency with which the particular emotion had been felt over the past week [11]. For example, ‘I found it difficult to work up the initiative to do things’ and ‘I felt that I was using a lot of nervous energy’. The scores are tallied according to the respective sub-scales (Depression, Anxiety or Stress). As the DASS – 21 is a short form version of the DASS −42, the subscale scores were doubled per the DASS −21 manual prior to categorisation and analysis. Symptom severity ratings for each sub-scale were assigned, ranging from normal to extremely severe, indicating the severity of symptoms [11] (Table 1). The DASS- 21 has a number of strengths and was chosen because it is validated for in and out of hospital use and has been proven to be a reliable screening tool for symptoms of depression, anxiety and stress, a feature unique to the DASS −21 and DASS – 42 [11,12]. Further, it does not require specialist training to administer [11,12]. The DASS- 21 has been widely used in both clinical and non-clinical samples including the injured population [11-13]. The DASS −21 does not give as full detail about precise symptoms as the DASS −42 but has equal factor structure [10].

**Table 1:** **Depression anxiety stress scale scores and clinical severity ratings**

**Ratings**	**Depression**	**Anxiety**	**Stress**
Normal	0-9	0-7	0-14
Mild	10-13	8-9	15-18
Moderate	14-20	10-14	19-25
Severe	21-27	15-19	26- 33
Extremely Severe	28+	20+	34+

### Data collection

Participants were approached within fourteen days of their admission or extubation. An interpreter was used to approach non-English speaking patients (n = 24). Following written consent the baseline DASS-21 was administered face to face by a researcher trained in structured interview technique and in managing potential participant distress. This interview was conducted at the patients’ bedside while they were admitted to hospital [14]. Although the DASS-21 tool does not require specialist training for clinicians, in the context of the research training was deemed important to maintain consistency of administration and for supporting participant wellbeing. The 3 and 6 month interviews were conducted via telephone following a structured telephone interview guide [14]. All participants were given contact details for follow up emotional support services.

### Data management

For data analysis a number of clinical categories were created. Polytrauma was defined as the patient having more than two injuries. This definition has been used widely in trauma research and is associated with subsequent long-term recovery [15] and higher treatment costs. Patients were grouped according to injury severity scores (ISS) of less than 9 (minor), 9–12 (moderate) and greater than 12 (major) [15]. For descriptive analyses, Depression, Anxiety and Stress scores were grouped in 3 categories: normal, mild/moderate, and severe/extremely severe as the aim of this research was to determine the incidence and severity of these reported symptoms. Hence, for analysis of predictors of depression, anxiety and stress, if a participant had a moderate, severe or extremely severe score in any of the depression, anxiety and stress subscales, they were allocated to a moderate and above group. An additional categorical variable was created using classification information for the moderate and above groups at baseline and at 3 months to capture the extent in which depression, anxiety and stress was experienced post injury. The categories were: Normal (did not experience moderate and above levels of depression, anxiety and stress scores in any of the 3 sub-scales at baseline and at 3 months); Baseline only (experienced moderate and above level of depression, anxiety and stress scores in any of the 3 sub-scales at baseline but not at 3 months); Persistent (experienced moderate and above level of depression, anxiety and stress score in any of the 3 sub-scales at baseline and at 3 months); Late onset (did not experience moderate and above level of depression, anxiety and stress score in any of the 3 sub-scales at baseline but experienced moderate and above level of depression, anxiety and stress score in any of the 3 sub-scales at 3 months). Dummy variables were subsequently created for these categories for the logistic regression with “normal” as the reference group.

### Data analysis

Data were analysed using SPSS 20 [16]. Descriptive statistics were conducted to determine sample representativeness in variables such as Mechanism of Injury, age and Injury Severity of the study participants and the eligible population over the 14-month data collection period. Descriptive statistics were also conducted to determine the incidence of depression, anxiety and stress at each time period. Pairwise comparisons were made between the two DASS-21 rating groups (moderate, severe or extremely severe versus normal and mild) to identify if any injury or demographic characteristic was associated with the levels of depression, anxiety and stress at each time point. Depending on the nature of the variable, the following statistical tests were used: T-test was used for the numeric age variable; Mann–Whitney test was used for ISS; χ^2^ test was used for categorical variables Polytrauma, Intensive Care Unit (ICU) Admission, and Injury mechanism.

Logistic regression was conducted to investigate factors that were associated with participants still experiencing a moderate or above level of depression, anxiety and stress 6 months after injury adjusting for other variables in the model with the intention of identifying significant predictors of the outcome (DASS) at 6 months post injury. These factors included whether participants sustained polytrauma as part of the injury, if they were admitted to ICU during their hospitalization for their injury, and whether they reported symptoms of depression, anxiety and stress post injury at either baseline or 3 months after injury (i.e. normal, baseline only, persistent, late onset). These variables were chosen to be included in the logistic regression based on their significant bivariate association with the reported levels on depression, anxiety and stress at 6 months at a significance of p < 0.05 level.

## Results

During the study period, 1335 patients were screened and 1024 met study criteria. Of these, 823 declined to participate. Participants who volunteered reasons for declining included not wanting to be involved in a longitudinal study or that they did not feel comfortable providing contact details for follow up. The demographic and injury characteristics of the study sample were representative of the general trauma population at the study site during the recruitment period in respect to age and injury severity. The majority of the sample population were male (74.6%) compared with 67.4% in the admitted trauma patient population which is expected and representative of the wider Australian major trauma population [17]. There were two main areas where there were differences in the study sample and the non -recruited admitted trauma patient population. Fewer patients in the sample group (10.4%) had an intensive care unit (ICU) admission compared to the non-recruited trauma patient population (17.5%) (p = 0.02), which is expected given the exclusion criteria. There was also a difference in the proportions of injury mechanism between the two groups, however all mechanisms were represented and this is not considered clinically significant (Table 2).

**Table 2:** **Sample representativeness of the study population**

**Characteristics**	**Total**	**Participants**	**Non-participants**	**Stat**
Patient demographics				
Total number of patients (n)	1335	201 (15.1%)	1134 (84.9%)	
Age (years)				
Mean (SD) [Range]	49 (23) [1–96]	49 (18) [18–94]	49 (24) [1–96]	T = 0.09, df = 345.7, p = 0.929
Gender (%)				χ^2^ = 4.16, df = 1, p = 0.041
Female	421 (31.5%)	51 (25.4%)	370 (32.6%)	
Male	914 (68.5%)	150 (74.6%)	764 (67.4%)	
Clinical characteristics				
ISS				
Median (IQR)	8 (4–16)	9 (5–14)	8 (4–16)	MW = 108948.0, p = 0.318
Poly trauma (%)				χ^2^ = 3.04, df = 1, p = 0.081
Yes	878 (65.8%)	143 (71.1%)	735 (64.8%)	
No	457 (34.2%)	58 (28.9%)	399 (35.2%)	
ICU admission (%)				χ^2^ = 5.42, df = 1, p = 0.020
Yes	181 (16.4%)	18 (10.4%)	163 (17.5%)	
No	921 (83.6%)	155 (89.6%)	766 (82.5%)	
Mechanism of injury				χ^2^ = 50.70, df = 10, p < 0.001
Fall greater than 1 Meter	464 (35.1%)	56 (27.9%)	408 (36.4%)	
MVC driver	262 (19.8%)	44 (21.9%)	218 (19.4%)	
MBC rider	114 (8.6%)	32 (15.9%)	82 (7.3%)	
Pedal cyclist	77 (5.8%)	23 (11.4%)	54 (4.8%)	
Violence	72 (5.4%)	10 (5%)	62 (5.5%)	
Pedestrian	114 (8.6%)	8 (4%)	106 (9.5%)	
MVC passenger	85 (6.4%)	8 (4%)	77 (6.9%)	
Industrial	27 (2%)	8 (4%)	19 (1.7%)	
MBC passenger	12 (0.9%)	4 (2%)	8 (0.7%)	
Penetrating or cutting injury	43 (3.3%)	3 (1.5%)	40 (3.6%)	
Sport and other	52 (3.9%)	5 (2.5%)	47 (4.2%)	

Of the study participants, one participant required a language translator. The mean age of participants was 49 years. The majority of the participants were male (74.6%) and the median ISS was 9 (IQR 5–14). The injury mechanism most represented was fall greater than 1 metre (27.9%), followed by motor vehicle collision (MVC) driver (21.9%) and motorbike collision (MBC) (15.9%). More than two thirds (71.1%) suffered polytrauma and 10.4% had an ICU admission. Of the 201 participants who completed the baseline interview, 122 (60.7%) completed the 3-month interview and 109 (54.2%) completed the 6 month interview.

### Incidence and severity of depression, anxiety and stress

The mean scores of reported depression, anxiety and stress reduced over time (Table 3). At baseline, 74 (36.8%) participants reported symptoms of depression above the normal range, 118 (58.7%) had symptoms of anxiety above the normal range and similarly, 89 (54.3%) had symptoms of stress above the normal range. Of these, 32 (15.9%) reported extremely severe scores in the depression subscale, 45 (22.4%) in anxiety, and 36 (17.9%) in stress.

**Table 3:** **Depression anxiety stress levels in trauma patients at baseline, 3 months, and 6 months post injury**

**Time point**	**Rating**	**Depression**	**Anxiety**	**Stress**
Baseline	Mean (SD)	9.4 (9.8)	10.3 (8)	14.5 (10.6)
	Normal	127 (63.2%)	83 (41.3%)	112 (55.7%)
	Mild- Moderate	42 (20.9%)	73 (36.3%)	53 (26.4%)
	Severe- Extremely Severe	32 (15.9%)	45 (22.4%)	36 (17.9%)
	Total participants with above normal rating	36.8%	58.7%	44.3%
3 months	Mean (SD)	9.3 (9.6)	6.4 (8)	12.8 (10)
	Normal	73 (59.8%)	80 (65.6%)	77 (63.1%)
	Mild- Moderate	30 (24.6%)	21 (17.2%)	29 (23.8%)
	Severe- Extremely Severe	19 (15.6%)	21 (17.2%)	16 (13.1%)
	Total participants with above normal rating	49 (40.2%)	42 (34.4%)	45 (36.9%)
6 months	Mean (SD)	5.9 (7.7)	4.2 (5.1)	8.9 (7.9)
	Normal	83 (76.1%)	85 (78%)	84 (77.1%)
	Mild- Moderate	21 (19.3%)	20 (18.3%)	21 (19.3%)
	Severe- Extremely Severe	5 (4.6%)	4 (3.7%)	4 (3.7%)
	Total participants with above normal rating	26 (23.9%)	24 (22.0%)	25 (22.9%)

Of the participants who completed the interview 3 months post injury, 49 (40.2%) had symptoms of depression above the normal range, 42 (34.4%) had symptoms of anxiety above the normal range and 45 (36.9%) had symptoms of stress that were above the normal range. Of these, 19 (15.6%), reported severe to extremely severe symptoms of depression, 21 (17.2%) in anxiety and 16 (13.1%) reported severe to extremely severe symptoms of stress.

At 6 months after injury, 26 (23.9%) of participants reported symptoms of depression that above the normal range, 24 (22.0%) reported symptoms of anxiety above the normal range and 25 (23.0%) reported symptoms of stress above the normal range. Of these participants 5 (4.6%), 4 (3.7%) and 4 (3.7%) reported severe to extremely severe symptoms of depression, anxiety and stress respectively (Table 3).

### Characteristics of participants with moderate and above levels of depression, anxiety and stress

The mean age of participants reporting moderate and above levels of depression, anxiety and stress at baseline, 3 and/or 6 months remained similar (48–50 years) with no particular age group reporting a significant increase or decrease in depression anxiety or stress scores at any one time point. Injury severity, mechanism of injury and gender also remained consistent across the time period (Table 4). Participants who had been admitted to ICU were more likely to report moderate and above level of depression, anxiety and stress at 3 (p = 0.03) and 6 months (p = 0.04) than those who were not admitted (Table 5).

**Table 4:** **Characteristics of patients with high depression, anxiety and stress levels at baseline, 3 and 6 months**

**Characteristics**	**Baseline DASS-21**	**Baseline DASS-21**	**Stat**	**3 month DASS-21**	**3 month DASS-21**	**Stat**	**6 month DASS-21**	**6 month DASS-21**	**Stat**
	**Moderate and above any subscale**	**Normal or mild any subscale**		**Moderate and above any subscale**	**Normal or mild any subscale**		**Moderate and above any subscale**	**Normal or mild any subscale**	
No. participants	114	87		50	73		28	81	
Mean age (yrs)^^^ (SD) [Range]	50 (18) [18–89]	48 (18) [17–94]	p = 0.32	48 (17) [20–88]	52 (16) [21–94]	p = 0.20	50 (17) [20–82]	53 (16) [23–94]	p = 0.36
Gender*									
*Female*	34 (29.8%)	17 (19.5%)	p = 0.97	15 (30%)	16 (21.9%)	p = 0.31	8 (28.6%)	19 (23.5%)	p = 0.59
*Male*	80 (70.2%)	70 (80.5%)		35 (70%)	57 (78.1%)		20 (71.4%)	62 (76.5%)	
Injury Severity Score (ISS) median (IQR)^+^	8 (4–14)	9 (5–14)	p = 0.62	9 (5–17)	8 (5–13)	p = 0.37	9.5 (4–21.5)	9 (5–13)	p = 0.55
Polytrauma*									
*Yes*	77 (67.5%)	66 (75.9%)	p = 0.20	40 (80%)	52 (71.2%)	p = 0.27	16 (57.1%)	64 (79%)	p = 0.02
*No*	37 (32.5%)	21 (24.1%)		10 (20%)	21 (28.8%)		12 (42.9%)	17 (21%)	
Intensive Care Unit (ICU) admission*									
*Yes*	13 (11.4%)	6 (6.9%)	p = 0.28	9 (18%)	4 (5.5%)	p = 0.03	6 (21.4%)	6 (7.4%)	p = 0.04
*No*	101 (88.6%)	81 (93.1%)		41 (82%)	69 (94.5%)		22 (78.6%)	75 (92.6%)	
Injury mechanism*									
Fall > 1 metre	32 (28.1%)	24 (27.6%)	p = 0.18	14 (28%)	18 (24.7%)	p = 0.08	9 (32.1%)	21 (25.9%)	p = 0.27
MVC driver	31 (27.2%)	13 (14.9%)		10 (20%)	16 (21.9%)		8 (28.6%)	16 (19.8%)	
MBC rider	17 (14.9%)	15 (17.2%)		9 (18%)	12 (16.4%)		6 (21.4%)	14 (17.3%)	
Pedal cyclist	8 (7%)	15 (17.2%)		3 (6%)	16 (21.9%)		0 (0%)	15 (18.5%)	
Violence	4 (3.5%)	19 (21.8%)		5 (10%)	0 (0%)		2 (7.1%)	2 (2.5%)	
Pedestrian	5 (4.4%)	6 (6.9%)		3 (6%)	1 (1.4%)		0 (0%)	3 (3.7%)	
MVC passenger	3 (2.6%)	5 (5.7%)		2 (4%)	1 (1.4%)		1 (3.6%)	2 (2.5%)	
Industrial	5 (4.4%)	3 (3.4%)		1 (2%)	5 (6.8%)		2 (7.1%)	3 (3.7%)	
MBC passenger	4 (3.5%)	0 (0%)		1 (2%)	1 (1.4%)		0 (0%)	2 (2.5%)	
Penetrating	2 (1.8%)	1 (1.1%)		1 (2%)	1 (1.4%)		0 (0%)	0 (0%)	
Other	3 (2.6%)	2 (2.3%)		1 (2%)	2 (2.7%)		0 (0%)	3 (3.7%)	

*Chi-Square test ^+^Mann–Whitney U test ^^^t-test.

**Table 5:** **Bivariate relationship between predictors and high levels of depression, anxiety and stress at 6 months post injury**

**Variables**	**Test statistics**	**Significance**
High levels of depression anxiety or stress	χ^2^ = 9.600, df = 3	p = 0.022
Age	T = 0.925, df = 107	p = 0.357
Gender	χ^2^ = 0.292, df = 1	p = 0.590
ISS	Mann–Whitney	p = 0.554
Polytrauma	χ^2^ = 5.096, df = 1	p = 0.024
ICU admission	χ^2^ = 4.175, df = 1	p = 0.041
Injury mechanism	χ^2^ = 11.039, df = 9	p = 0.273

### Predictors of high depression anxiety and stress at 6 months post injury

Participants who had been admitted to ICU were 4 times as likely to report moderate or above level of depression, anxiety and stress in any of the 3 DASS-21 subscales at 6 months post injury when compared to those who had not been admitted (OR 4.440, CI [1.109- 17.767]). Participants who reported moderate or above levels of depression, anxiety and stress at baseline and 3 months (persistent) post injury were nearly 3 times as likely to report moderate or above levels of depression, anxiety and stress at 6 months post injury (OR 3.071, CI [1.050- 8.988]) when compared to patients who did not experience any elevated levels of depression, anxiety and stress at all. Participants who experienced late onset of depression, anxiety and stress (normal at baseline but moderate and above at 3 months were nearly 6 times as likely to report psychological distress at 6 months following injury when compared to those who did not experience any elevated levels of depression, anxiety and stress at all (OR 5.896, CI [1.390- 25.031]) (Table 6).

**Table 6:** **Predictors for moderate or above depression anxiety or stress scores in any subscale at 6 months post injury**

**Variables in logistic regression**	**Odd ratios**	**95% C.I**	**Sig.**
Polytrauma	0.191	0.065 – 0.562	0.003
ICU admission	4.440	1.109 – 17.767	0.035
Persistent (mod/above depression anxiety or stress at baseline and 3 months)	3.071	1.050 – 8.988	0.041
Late onset (mod/above depression anxiety or stress first reported at 3 months)	5.896	1.390 – 25.013	0.016

Model Significance: χ^2^ = 19.237.19, df = 4, p = 0.001; Nagelkerke R^2^ = 0.238.

Hosmer-Lemeshow goodness of fit statistic: χ^2^ = 3.017, df = 4, p = 0.555.

## Discussion

In this study, depression, anxiety and/or stress occurred following injury in a high proportion of patients during hospitalization (58.7%), and at 3 (40.2%) and 6 months following injury (23.9%). The severity of these symptoms ranged from mild to extremely severe.

The factor found to be primarily associated with the incidence of depression, anxiety and stress symptoms at three-month post injury in this study was an Intensive Care Unit admission. The factors associated with depression, anxiety and stress symptoms six months post injury were again, an Intensive Care Unit admission, and secondly, the reporting of depression, anxiety and/or stress symptoms in the moderate/severe/extremely severe categories at 3 months post injury.

Although ICU admission was a predictor of persistent depression, anxiety and stress symptoms, all patients admitted to hospital following injury should be screened as the majority of participants reporting moderate and above depression, anxiety and stress symptoms were not severely injured and may not have had an ICU admission. These findings are consistent with other literature describing the mental health outcomes in both the injured [17-20] and non-injured population [18-20]. The finding that a large proportion of participants had high depression, anxiety and stress scores at 3 months suggests routine longitudinal follow-up is needed and supports findings by Richmond et al., [21] who showed that injured participants experience an increase in psychological distress at 3 months after discharge from hospital when compared to baseline findings. The finding that ISS and polytrauma is not a predictor of depression, anxiety and stress after injury is also consistent with the existing evidence [22-24].

The study findings support the use of the DASS-21 in the injured population and the findings indicate that patients require mental health follow up post injury. There is a known lack of mental health follow up post discharge from hospital following injury [7]. The process for routine screening and related resource implications needs to be considered. If patients attend a trauma outpatient clinic for routine follow up they could be screened by the treating clinician or a clinic nurse in addition to their clinical follow up after injury. Or, if in hospital resources are limited, at the very least patients who have had an ICU admission should be targeted.

As a result of this study, in February 2014 the study site commenced screening patients for mental health sequelae using the DASS-21 in the trauma outpatient clinic in conjunction with their clinical follow up. A process for referral of patients reporting symptoms to clinical psychologists and/or in cases of active suicidality to the site acute mental health team was developed by the clinic trauma nurse in consultation with the site mental health team. Over ten patients requiring referral for mental health intervention have been identified through this process, which is being monitored for potential further evaluation. This does, however, exclude patients who do not return to the clinic for review who may also be at risk. To mitigate this, in all patient discharge letters, general advice about the emotional sequelae of injury is now included in conjunction with instruction to follow up with their General Practitioner or the Trauma outpatient clinic should they experience symptoms of depression, anxiety and stress post injury. The development of a standardized discharge information pack, informed by the study findings is under development, which includes information regarding symptom recognition, risk factors and resources for follow-up.

Routine in-hospital screening and follow up would promote anticipatory guidance for care and this is essential for the holistic wellbeing and health outcomes of the injured patient. The early identification of symptoms of depression, anxiety and stress post-injury would facilitate early intervention. Early intervention for mental health in general may also prevent the progression of Acute Stress Disorder symptoms to Post Traumatic Stress Disorder [25], particularly as anxiety symptoms may be correlated with the development of PTSD symptoms [26].

Future research could include the relationship between DAS and PTSD in this clinical population, the presence of co-morbid physical and mental illness and ongoing physical illness following hospital discharge. Qualitative exploration to explain the study findings would assist in understanding the role that physical and psychosocial factors relating to injury play in the development of negative emotion states, and the factors that may prevent or ameliorate these injury-related impacts. The significance of social support for the injured patient, for example, has been previously identified [27].

The limitations of this study included the small sample size and loss to follow up making it impossible to exclude the possibility that these participants did not have ongoing symptoms of depression, anxiety and stress. The study also attracted a small response rate. For example, there were significantly fewer patients who required an ICU admission in the study group than in the non study group, this could represent an under reporting of the magnitude of the incidence of elevated depression, anxiety and stress given that an ICU admission was a predictor of above normal levels. This group could also have been precluded from participating due to the severity of their cognitive impairment following traumatic brain injury. Further, there was no reliable recorded patient data on pre-existing mental health diagnosis, resulting in the inability to acknowledge pre-existing symptoms. Nor was the misuse of alcohol and other drugs recorded, which are known factors associated with the symptoms of depression and anxiety [28].

## Conclusion

Depression, anxiety and stress symptoms occur following injury in a high proportion of patients during hospitalization, and at 3 and 6 months following injury, particularly those requiring intensive care. The DASS-21 has the ability to identify longer-term symptoms of depression, anxiety and stress in injured patients. All injured patients would benefit from routine mental health screening, particularly at 3 months following injury.
